# Modeling and managing household travels taking into considering of school bus

**DOI:** 10.1371/journal.pone.0324561

**Published:** 2025-05-29

**Authors:** Qing Dai, Jiajia Zhang

**Affiliations:** 1 School of Information Engineering, Dalian Ocean University, Dalian, China; 2 School of Maritime Economics and Management, Dalian Maritime University, Dalian, China; The University of Tokyo, JAPAN

## Abstract

This paper examines the household commuting problem in a bi-modal transportation that includes both autos and school buses. Traditional studies of household commuting have assumed that adults first drop off their children at school before driving to work. However, with the increasing use of school buses in metropolitan areas, household commuting patterns have changed. This paper investigates the impact of school buses on household travel behavior during morning peak hours. Simultaneously, this paper also takes into account the positive utilities of household commuters’ activities at home, school and workplace, as well as the negative utilities of travel time and schedule delay. In this paper, based on activity approach, first, a net utility function is developed using these factors. Then, based on the net utility function, the occurrence conditions of all the possible equilibrium travel patterns are analytically solved and the properties of the user equilibrium are researched. Specifically, this paper examines how equilibrium travel patterns are affected by school bus fares and school-work start time difference, and at equilibrium travel patterns, the net utilities of household commuters are also analyzed. Finally, a first-best time-varying toll model is suggested to alleviate traffic congestion caused by household commuting.

## Introduction

The widespread popularity of autos has led to serious traffic congestion in many metropolitan areas, which causes both economic losses and environmental pollution. To address this issue, numerous researchers [1–6] have focused on managing traffic congestion during morning peak hours. One of the seminal contributions to morning peak commuting was made by Vickrey [7], who proposed a classic bottleneck model that remains the basis for studying morning peak commuting. In his model, a single bottleneck with a fixed capacity or service rate is on the road between home and workplace. Every morning, a fixed number of homogeneous commuters must pass through the bottleneck to reach their workplaces. At the bottleneck, if the arrival rate exceeds its service rate, a queue will be formed, that is, not all commuters can arrive at their workplaces on time. In this context, commuters must trade off queuing time against schedule delay to minimize their travel costs. Equilibrium is achieved when all commuters are unable to further reduce their travel costs by changing their departure times. Vickrey’s model has therefore produced several model extensions to address a series of issues, such as user heterogeneity [8–10], road pricing [11,12], tradable credits [13,14], carpooling or carsharing [1,15,16], stochastic travel demand and/or bottleneck capacity [17–19]. More details can be found in [20].

Most previous research on morning peak commuting issues has focused primarily on individual travelers [21–25], however, during morning peak hours, most commuters are household travelers [26]. Compared with individual commuters, household travelers must consider travel cost of all household members with travel needs when choosing their departure time. For the particularity of household travelers, Jia et al. [26] developed a model to study their departure time choice behavior and analyzed equilibrium trip scheduling. Moreover, a one-step toll strategy and a tradable credit scheme were provided to manage household commuting. Liu et al. [27] and Zhang et al. [28] studied the mixed travel patterns, including both household and individual travelers, during travel congestion in morning commute. Their results shown that at user equilibrium with mixed travelers, the dynamic departure pattern varies with the ratio of individual travelers to household travelers, as well as the school-work start time difference, and they found total travel cost can be reduced by appropriately coordinating the schedule gap. He et al. [29] examined mixed travel patterns in a Y-shape network with two bottlenecks, considering the impact of the school-work start time difference, as well as bottleneck capacity expansion on social welfare. Their results shown that the optimal staggering policy on system performance depends on the demand distribution of the two groups. Dai et al. [30] investigated the morning commute problem for household travelers with different activities, such as school travel (home-school-home) and household travel (home-school-work). They concluded that adjusting the school-work start time difference could reduce total system travel costs. Li et al. [31] analyzed the impact of bottleneck capacity expansion on households of different income levels under various congestion pricing strategies. Their results shown that the benefits of bottleneck capacity expansion for households of different income levels vary depending on the pricing scheme. Zhang et al. [32] examined the impact of parking space constraints and parking reservation mechanisms on the morning commute of household travelers in a two-mode transport system, where travelers use either cars or rail. Their study concluded that appropriate parking reservation allocation and a suitable school-work time difference can minimize total travel costs, reduce congestion, and improve social welfare. Li et al. [33] conducted a comparative analysis of the impact of school locations on the commuting problem during the morning peak period in a Y-shaped road network, and examined the welfare effects of staggered school and work start times. Their results indicated that the location of schools significantly influences policy performance, and that, under certain conditions, staggering school and work start times can simultaneously improve the welfare of both individual and household commuters. However, most existing studies on household travel focus primarily on scenarios where household members share a single vehicle, limiting their applicability to more diverse commuting patterns. In contrast, this study expands upon the existing literature by considering the broader dynamics of household travel, including the interactions between household members with varying travel needs and the implications these interactions have on departure time and mode choice. In contrast to previous works, which mainly examine single-vehicle households or individual vehicle use, our model accounts for the collective decision-making process in households with multiple travelers. By incorporating both private vehicles and school buses, this study provides a more comprehensive understanding of household commuting behavior and its impact on congestion during the morning peak period.

With the increasing popularity of school buses in metropolitan areas, this transportation mode will change household commuting patterns. Consequently, households must consider both their departure time and the travel mode in the morning. Moreover, the existing models [22,26,27] for household travel only consider bottleneck congestion and schedule delays, neglecting the motivations for trips, temporal constraints, and dependencies of activities in destination. These omissions may lead to inaccurate guidance for the formulation of transport policy [34]. For example, Li et al. [34] proposed an activity-based bottleneck model and their research showed that departure time of commuters will be incorrectly estimate if the impact of activity utility on commuters is not considered. Kim and Kwan [35] analyzed the crowdsourced real-time traffic congestion data and activity-travel data of 250 individuals in Los Angeles, and they found that ignoring the impact of activities on travel may lead to significantly underestimating traffic congestion. Therefore, based on the impact of activities, this study investigates household commuting issues in a bi-modal transportation system that includes both autos and school buses. Different from previous studies of household commuting, this study considers two groups of household travelers, namely Group 1 and Group 2. In Group 1, household travelers need to drop off their children at school before heading to work, while in Group 2, adults of household travelers drive themselves to work and their children go to school by school bus. Each household traveler must choose their departure time and transportation mode every morning to maximize their utility. Equilibrium is achieved when no one can increase their utility further by changing their departure time or transportation mode.

The contributions of this paper are threefold. First, based on the model developed by Jia et al. [26], we extend their framework by incorporating two transportation modes and considering the activity utilities of household travelers at home, school, and workplace. Second, we identify all equilibrium travel patterns and analyze the properties of these equilibria. Finally, we derive the first-best time-varying toll for eliminating congestion externalities. Our findings show that when the total number of household travelers remains constant, the ratio of the two groups of household travelers is determined endogenously by the school bus fare. Additionally, we identify seven equilibrium patterns, which are influenced by the school bus fare and the school-work start time difference.

The remainder of this paper is organized as follows. Section 2 introduces the problem description and the model. Furthermore, the traffic patterns at departure equilibrium with two groups of household travelers are analyzed. In Section 3, the impacts of school bus fare, work start time and school start time on the user net utility are examined. Section 4 provides a first-best time-varying toll strategy for eliminating travel congestion caused by the bottleneck. Section 5 provides numerical illustration and verification. Last section presents some conclusions of this study.

## Problem description and model formulation

**Fig 1:**
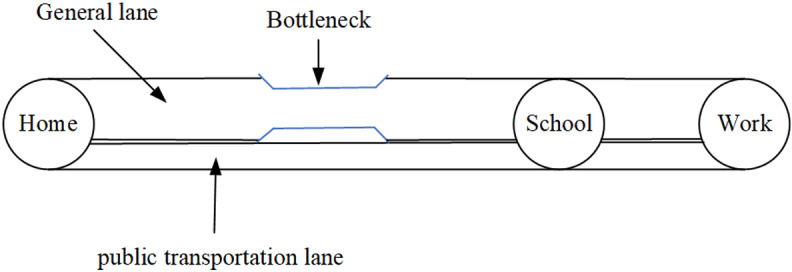
City network.

This paper examines a network consisting of a public transportation lane and a general lane, as illustrated in Fig 1. During morning peak hours, autos use the general lane while school buses use the public transportation lane. A bottleneck with a fixed capacity (s) is located between school and home. There are two groups (Group 1 and Group 2) of commuters, that is, in Group 1, adults of household travelers first send their children to school and then drive to work, while in Group 2, adults of household travelers have their children take school bus to school and they drive directly to work. The school start time and the work start time are denoted by $t_{s}^{*}$ and $t_{w}^{*}$, respectively. Simultaneously, the situation where work starts later than school, i.e., $t_{s}^{*}\leq t_{w}^{*}$, is considered. $\Delta t=t_{w}^{*}-t_{s}^{*}$ represents the difference between the work start time and the school start time.

### Household travelers in Group 1

In Group 1, the net utility includes the negative travel utility (travel time cost and schedule delay costs) for adults and children, as well as the positive utility of activities (home, school and workplace activity).

The negative travel utility for household travelers in Group 1 can be expressed as:


$$\begin{array}{cc} C_{1}(t) & =\alpha T_{w}(t)+\beta max\{0,\left({t_{w}}^{*}-t-T_{w}(t))\right\}+\gamma max\left\{0,\left(t+T_{w}(t)-{t_{w}}^{*}\right)\right\}\\ & +\alpha T_{s}(t)+\beta max\{0,\left({t_{s}}^{*}-t-T_{s}(t))\right\}+\gamma max\left\{0,\left(t+T_{s}(t)-{t_{s}}^{*}\right)\right\}, \end{array}$$ (1)

where α represents the unit cost of travel time, and β and γ represent the unit costs of arriving early and late, respectively. It is assumed that γ>2α>2β [26]. The travel time of adults and children at departure time t are denoted by $T_{w}(t)$ and $T_{s}(t)$, respectively, and can be expressed as


$$T_{w}(t)=T^{fw}+T(t),$$ (2)

$$T_{s}(t)=T^{fs}+T(t),$$ (3)

where $T^{fs}$ and $T^{fw}$ represent the fixed free-flow travel time from home to school and from home to workplace, respectively. Without loss of generality, $T^{fs}=0$ and $T^{fw}=0$ are assumed [26]. Thus, $T_{w}(t)=T_{s}(t)=T(t)$ is obtained. Eq. (1) can be reformulated as


$$\begin{array}{cc} C_{1}(t) & =2\alpha T(t)+\beta max\{0,\left({t_{w}}^{*}-t-T(t))\right\}+\gamma max\left\{0,\left(t+T(t)-{t_{w}}^{*}\right)\right\}\\ & +\beta max\{0,\left({t_{s}}^{*}-t-T(t))\right\}+\gamma max\left\{0,\left(t+T(t)-{t_{s}}^{*}\right)\right\}. \end{array}$$ (4)

In equation (4), we assume identical schedule early/delay costs for both adult and child household members to maintain model simplicity and avoid unnecessary complexity. While we recognize that in reality, there could be differences between adults and children in terms of flexibility and time constraints. Future research might address this issue, along with considering a more general user heterogeneity among different groups of travelers. Let D(t) denote the length of the queue at time t. It is defined as the difference between the cumulative arrival rate and the cumulative departure rate at the bottleneck. Therefore, D(t) can be expressed as


$$D(t)=\int_{\hat{t}}^{t}r(u)du-s\left(t-\hat{t}\right),$$ (5)

where $\hat{t}$ denotes the most recent time at which there was no queue, and r(t) represents the departure rate at time t. Then, queuing time T(t) can be expressed as


$$T(t)=\frac{D(t)}{s}.$$ (6)

A constant marginal-activity utility is assumed. Then, in Group 1, the positive utility of activities for household travelers can be expressed as


$$U_{1}(t)=2u_{h}\left(t-t_{-}\right)+\left(u_{s}+u_{w}\mathrm{\backslash rightleft}(t^{-}-t-T(t)\right),$$ (7)

where $t_{-}$ and $t^{-}$ denote the start time and the end time of morning peak commute, respectively, and $u_{h}$, $u_{s}$ and $u_{w}$ represent the unit utility of activities at home, school and workplace, respectively. Assuming adults and children have the same $u_{h}$. Additionally, it supposes that ${u_{w}>u}_{s}>u_{h}$, which ensures that household commuters have an incentive to travel during morning peak hours.

Based on the above analysis, in group 1, the net utility of household commuters can be expressed as follows:


$$\psi_{1}(t)=U_{1}(t)-C_{1}(t)=2u_{h}\left(t-t_{-}\right)+\left(u_{s}+u_{w}\mathrm{\backslash rightleft}(t^{-}-t-T(t)\right)-[2\alpha T(t)+\beta max\{0,\left({t_{w}}^{*}-t-T(t))\right\}+\gamma max\left\{0,\left(t+T(t)-{t_{w}}^{*}\right)\right\}+\beta max\{0,\left({t_{s}}^{*}-t-T(t))\right\}+\gamma max\left\{0,\left(t+T(t)-{t_{s}}^{*}\right)\right\}].$$ (8)

Due to $t_{s}^{*}\leq t_{w}^{*}$, Eq. (8) can be expressed as a piecewise function:


$$\begin{array}{c} \psi_{1}(t)=\left\{ \begin{array}{c} \begin{array}{cc} \begin{array}{c} \left({2u}_{h}-u_{s}-u_{w}+2\beta \right)t-\left(u_{s}+u_{w}+2\alpha -2\beta \right)T(t)-\\ \beta \left({t_{w}}^{*}+{t_{s}}^{*}\right)-{2u}_{h}t_{-}+\left(u_{s}+u_{w}\right)t^{-}, \end{array} & t+T(t)<{t_{s}}^{*} \end{array}\\ \begin{array}{cc} \begin{array}{c} \left({2u}_{h}-u_{s}-u_{w}+\beta -\gamma \right)t-\left(u_{s}+u_{w}+2\alpha -\beta +\gamma \right)T(t)-\\ \beta {t_{w}}^{*}+{\gamma t_{s}}^{*}-{2u}_{h}t_{-}+\left(u_{s}+u_{w}\right)t^{-}, \end{array} & {t_{s}}^{*}\leq t+T(t)\leq {t_{w}}^{*} \end{array}\\ \begin{array}{cc} \begin{array}{c} \left(2u_{h}-u_{s}-u_{w}-2\gamma \right)t-\left(u_{s}+u_{w}+2\alpha +2\gamma \right)T(t)+\\ \gamma \left({t_{w}}^{*}+{t_{s}}^{*}\right)-{2u}_{h}t_{-}+\left(u_{s}+u_{w}\right)t^{-}, \end{array} & t+T(t)>{t_{w}}^{*} \end{array} \end{array}\right.. \end{array}$$ (9)

As mentioned above, it can be known that $t_{s}^{*}\leq t_{w}^{*}$. Based on the research by Jia et al. (2016), the departure time of household commuters in Group 1 can be classified into three periods: i) early for both school and workplace; ii) late for school but early for workplace; iii) late for both school and workplace. According to the equilibrium condition $\frac{d\psi (t)}{dt}=0$, the departure rates for these three periods can be expressed as


$$\frac{d\psi_{1}(t)}{dt}=0\to \left\{ \begin{array}{c} r_{11}=\frac{{2u}_{h}+2\alpha }{u_{s}+u_{w}+2\alpha -2\beta }s,t+T(t)<{t_{s}}^{*}\\ r_{12}=\frac{2u_{h}+2\alpha }{u_{s}+u_{w}+2\alpha -\beta +\gamma }s,{t_{s}}^{*}\leq t+T(t)\leq {t_{w}}^{*}\\ r_{13}=\frac{2u_{h}+2\alpha }{u_{s}+u_{w}+2\alpha +2\gamma }s,t+T(t)>{t_{w}}^{*} \end{array}.\right.$$ (10)

Based on the departure rate provided by Eq. (10), the following proposition can be deduced.

**Proposition 1**. When all household commuters belong to Group 1, queueing occurs at the bottleneck if and only if ${2u}_{h}+2\beta >u_{s}+u_{w}$, otherwise there is no queuing.

**Proof**: To prove this proposition, we can compare the relationship between the departure rate and the capacity s at the bottleneck. Based on Eq. (10), it can be known that when ${2u}_{h}+2\beta \leq u_{s}+u_{w}$, $r_{13}<r_{12}<r_{11}\leq s$ is obtained. Therefore, the departure rates of all periods are less than the capacity s at the bottleneck, which means that there is no queuing before the bottleneck. However, when ${2u}_{h}+2\beta >u_{s}+u_{w}$, $r_{11}>s$ is obtained. When the departure rate exceeds the capacity s, then queuing will inevitably occur. Therefore, the proposition is proved.

Although there are three possible departure periods, however, not all three periods will occur simultaneously in dynamic traffic patterns. For instance, when Δt is large enough, whether the household travelers arrive at school early or late, they will arrive at workplace early. Thus, in this case, there are only two periods as follows: i) early for school and workplace; ii) late for school but early for workplace.

### Household travelers in Group 2

In Group 2, adults of household members drive to work while their children go to school by school bus. During morning peak hours, school buses can use the public transportation lane, so the children who use school buses do not experience traffic congestion. It is assumed that school buses can arrive at the school on time, that is, the children do not incur any schedule delay costs. Based on this analysis, the negative travel utility of household commuters in group 2 can be expressed as


$$C_{2}(t)=\alpha T(t)+\beta max\{0,\left({t_{w}}^{*}-t-T(t))\right\}+\gamma max\left\{0,\left(t+T(t)-{t_{w}}^{*}\right)\right\}+P,$$ (11)

where P is the fare of school bus.

Different from the household commuters in Group 1, children of household travelers in Group 2 do not experience traffic congestion and are able to arrive at school on time. Therefore, the positive utility of activities for household travelers in Group 2 can be expressed as


$$U_{2}(t)=u_{h}\left({t_{s}}^{*}-t_{-}\right)+u_{s}\left(t^{-}-{t_{s}}^{*}\right)+u_{h}\left(t-t_{-}\right)+u_{w}\left(t^{-}-t-T(t)\right),$$ (12)

where the first two items (namely, $u_{h}\left({t_{s}}^{*}-t_{-}\right)$ and $u_{s}\left(t^{-}-{t_{s}}^{*}\right)$) of Eq. (12) are the children’s activity utilities and the latter two items (namely, $u_{h}\left(t-t_{-}\right)$ and $u_{w}\left(t^{-}-t-T(t)\right)$) are the adult’s activity utilities. In addition, it can be seen from Eq. (12) that the children’s activity utility has nothing to do with departure time t.

According to Eq. (11) and Eq. (12), in Group 2, the net utility of household commuters can be expressed as


$$\psi_{2}(t)=U_{2}(t)-C_{2}(t)=u_{h}\left({t_{s}}^{*}-t_{-}\right)+u_{s}\left(t^{-}-{t_{s}}^{*}\right)+u_{h}\left(t-t_{-}\right)+u_{w}\left(t^{-}-t-T(t)\right)-\left[\alpha T(t)+\beta \max\{0,\left({t_{w}}^{*}-t-T(t))\right\}+\gamma max\left\{0,\left(t+T(t)-{t_{w}}^{*}\right)\right\}+P\right],$$ (13)

Due to $t_{s}^{*}\leq t_{w}^{*}$, the net utility can also be expressed as a piecewise function:


$$\psi_{2}(t)=\left\{ \begin{array}{c} \begin{array}{cc} \begin{array}{c} \left(u_{h}-u_{w}+\beta \right)t-\left(u_{w}+\alpha -\beta \right)T(t)-\beta {t_{w}}^{*}+\\ \left(u_{h}-u_{s}\right){t_{s}}^{*}-{2u}_{h}t_{-}+\left(u_{s}+u_{w}\right)t^{-}-P, \end{array} & t+T(t)<{t_{w}}^{*} \end{array}\\ \begin{array}{cc} \begin{array}{c} \left(u_{h}-u_{w}-\gamma \right)t-\left(u_{w}+\alpha +\gamma \right)T(t)+\gamma {t_{w}}^{*}+\\ \left(u_{h}-u_{s}\right){t_{s}}^{*}-{2u}_{h}t_{-}+\left(u_{s}+u_{w}\right)t^{-}-P, \end{array} & t+T(t)\geq {t_{w}}^{*} \end{array} \end{array}\right..$$ (14)

As $\frac{d\psi (t)}{dt}=0$, the departure rates of adults from home who reach the workplace before and after desired arrival time $t_{w}^{*}$ are expressed as follows:


$$\frac{d\psi_{2}(t)}{dt}=0\to \left\{ \begin{array}{c} r_{21}=\frac{u_{h}+\alpha }{u_{w}+\alpha -\beta }s,t+T(t)<{t_{w}}^{*}\\ r_{22}=\frac{u_{h}+\alpha }{u_{w}+\alpha +\gamma }s,t+T(t)\geq {t_{w}}^{*} \end{array}.\right.$$ (15)

According to Eq. (15), the following proposition are obtained.

**Proposition 2**. When all household commuters belong to Group 2, queueing occurs at the bottleneck if and only if $u_{h}+\beta >u_{w}$, otherwise there is no queuing.

**Proof**: The reasoning to verify Proposition 2 is similar to that for Proposition 1.

This paper focuses on the scenario where queueing occurs at the bottleneck, which means that the conditions for queuing at the bottleneck in Proposition 1 and 2 must be met simultaneously, that is, inequality $u_{h}+\beta >u_{w}>u_{s}$ must be satisfied. Moreover, based on Eq. (10) and Eq. (15), it can be known that the departure rates are sorted in the following order: $r_{11}>r_{21}>r_{12}>r_{13}>r_{22}$.

## Dynamic user equilibrium

**Fig 2:**
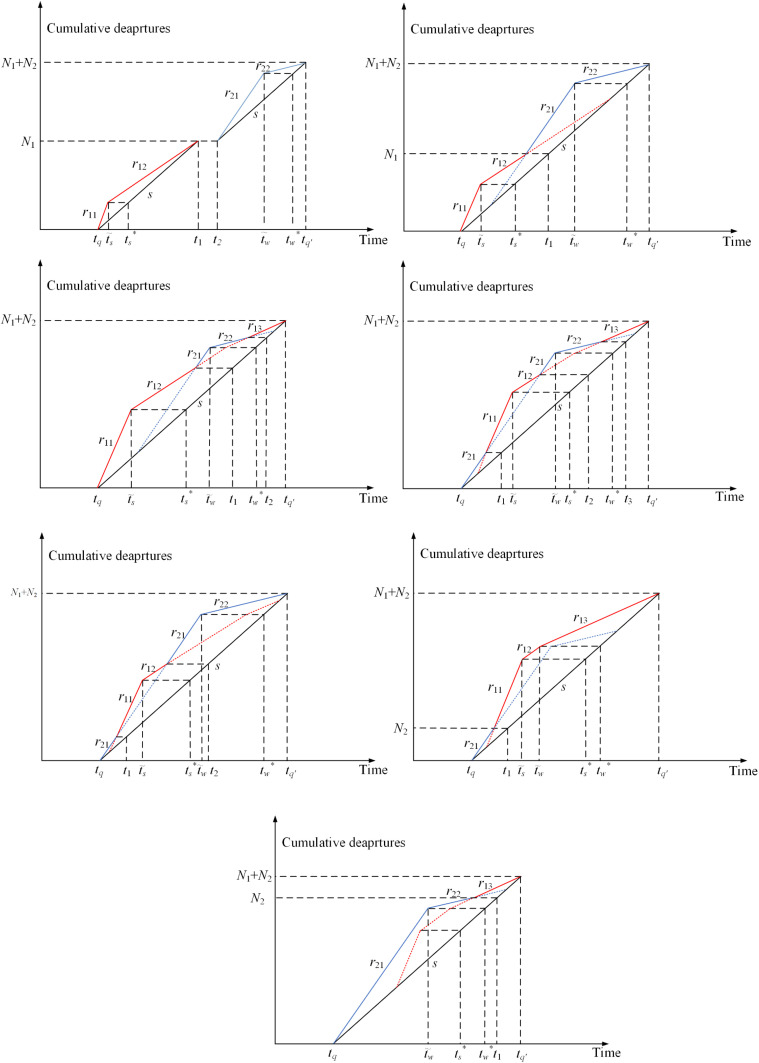
Seven possible equilibrium travel patterns. (a) Pattern **1**. (b) Pattern **2**. (c) **Pattern 3**. (d) **Pattern 4**. (e) **Pattern 5**. (f) **Pattern 6**. (g) **Pattern 7.**

Each household commuter aims to maximize their net utility by choosing their departure time and transportation mode. When no household commuter can unilaterally change their departure time or transportation mode to increase their net utility, the dynamic user equilibrium will be obtained. It is assumed that the total demand number N of household commuters is fixed, but the number of households in Group 1 ($N_{1}$) and Group 2 ($N_{2}$) is endogenous and can be controlled by school bus fare P. Based on Δt and P, seven possible equilibrium travel patterns can be derived as shown in Fig 2, and the occurrence conditions for each pattern are summarized in Table 1. The proofs of the occurrence conditions are provided in Appendix A. Additionally, Table 2 presents the critical time points for each equilibrium travel pattern. The equilibrium travel patterns are described as follows. Let ${\tilde{t}}_{s}$ be the departure time of household commuters who arrive at school on time, i.e., ${\tilde{t}}_{s}+T\left({\tilde{t}}_{s}\right)={t_{s}}^{*}$, and ${\tilde{t}}_{w}$ is the departure time of household commuters who arrive at workplace on time, i.e., ${\tilde{t}}_{w}+T\left({\tilde{t}}_{w}\right)={t_{w}}^{*}$. let $t_{q}$ and $t_{q^{\prime }}$ represent the start and end times of morning peak, respectively.

**Table 1:** The occurrence conditions of all possible equilibrium travel patterns.

Patterns	Conditions	Figs
1	$\Delta t\geq \frac{{\gamma -u}_{h}+u_{w}}{\beta +\gamma }\frac{N}{s}-\frac{u_{s}+2u_{w}-3u_{h}-2\beta +\gamma }{\left(u_{s}+u_{w}-2u_{h}-2\beta \mathrm{\backslash rightleft}(u_{h}-u_{s}-\gamma \right)}P$	(a)
2	$\Delta t<\frac{{\gamma -u}_{h}+u_{w}}{\beta +\gamma }\frac{N}{s}-\frac{u_{s}+2u_{w}-3u_{h}-2\beta +\gamma }{\left(u_{s}+u_{w}-2u_{h}-2\beta \mathrm{\backslash rightleft}(u_{h}-u_{s}-\gamma \right)}P$ $\Delta t\geq -\frac{{2u}_{h}-u_{s}-u_{w}+2\beta }{\beta +\gamma }\frac{N}{s}+\frac{2}{{u_{s}+\gamma -u}_{h}}P$ $\Delta t\geq \frac{u_{w}+\gamma -u_{h}}{\beta +\gamma }\frac{N}{s}-\frac{1}{u_{h}-u_{s}+\beta }P$	(b)
3	$\Delta t<\frac{{\gamma -u}_{h}+u_{w}}{\beta +\gamma }\frac{N}{s}-\frac{u_{s}+2u_{w}-3u_{h}-2\beta +\gamma }{\left(u_{s}+u_{w}-2u_{h}-2\beta \mathrm{\backslash rightleft}(u_{h}-u_{s}-\gamma \right)}P$ $\Delta t<-\frac{{2u}_{h}-u_{s}-u_{w}+2\beta }{\beta +\gamma }\frac{N}{s}+\frac{2}{{u_{s}+\gamma -u}_{h}}P$ $\Delta t\geq \frac{u_{s}+u_{w}+2\gamma -2u_{h}}{\beta +\gamma }\frac{N}{s}-\frac{2}{u_{h}-u_{s}+\beta }P$	(c)
4	$\Delta t<\frac{{\gamma -u}_{h}+u_{w}}{\beta +\gamma }\frac{N}{s}-\frac{u_{s}+2u_{w}-3u_{h}-2\beta +\gamma }{\left(u_{s}+u_{w}-2u_{h}-2\beta \mathrm{\backslash rightleft}(u_{h}-u_{s}-\gamma \right)}P$ $\Delta t<\frac{u_{s}+u_{w}+2\gamma -2u_{h}}{\beta +\gamma }\frac{N}{s}-\frac{2}{u_{h}-u_{s}+\beta }P$ $\Delta t<\frac{1}{u_{s}+\gamma {-u}_{h}}P+\frac{u_{w}-u_{h}-\beta }{\beta +\gamma }\frac{N}{s}$ $\Delta t\geq \frac{1}{\left(u_{s}+\gamma {-u}_{h}\right)}P+\frac{\left(-u_{h}+u_{w}-\beta \mathrm{\backslash rightleft}(2u_{h}-u_{s}-u_{w}-2\gamma \right)}{\left(u_{w}-u_{s}-2\beta -2\gamma \right)\left(u_{s}+\gamma {-u}_{h}\right)}\frac{N}{s}$ $\Delta t\geq \frac{\left(u_{w}-u_{s}-2\beta -2\gamma \right)}{(\beta +\gamma )\left(u_{h}-u_{w}+\beta \right)}P-\frac{2u_{h}-u_{s}-u_{w}-2\gamma }{\beta +\gamma }\frac{N}{s}$	(d)
5	$\Delta t<\frac{u_{w}+\gamma -u_{h}}{\beta +\gamma }\frac{N}{s}-\frac{1}{u_{h}-u_{s}+\beta }P$ $\Delta t\geq \frac{1}{u_{s}+\gamma {-u}_{h}}P+\frac{u_{w}-u_{h}-\beta }{\beta +\gamma }\frac{N}{s}$	(e)
6	$\Delta t<\frac{{\gamma -u}_{h}+u_{w}}{\beta +\gamma }\frac{N}{s}-\frac{u_{s}+2u_{w}-3u_{h}-2\beta +\gamma }{\left(u_{s}+u_{w}-2u_{h}-2\beta \mathrm{\backslash rightleft}(u_{h}-u_{s}-\gamma \right)}P$ $\Delta t<\frac{1}{\left(u_{s}+\gamma {-u}_{h}\right)}P+\frac{\left(-u_{h}+u_{w}-\beta \mathrm{\backslash rightleft}(2u_{h}-u_{s}-u_{w}-2\gamma \right)}{\left(u_{w}-u_{s}-2\beta -2\gamma \right)\left(u_{s}+\gamma {-u}_{h}\right)}\frac{N}{s}$ $\Delta t\geq \frac{\left(u_{w}-u_{s}-2\beta -2\gamma \right)}{(\beta +\gamma )\left(u_{h}-u_{w}+\beta \right)}P-\frac{2u_{h}-u_{s}-u_{w}-2\gamma }{\beta +\gamma }\frac{N}{s}$ $\Delta t<\frac{u_{s}+u_{w}+2\gamma -2u_{h}}{\beta +\gamma }\frac{N}{s}-\frac{2}{u_{h}-u_{s}+\beta }P$	(f)
7	$\Delta t<\frac{{\gamma -u}_{h}+u_{w}}{\beta +\gamma }\frac{N}{s}-\frac{u_{s}+2u_{w}-3u_{h}-2\beta +\gamma }{\left(u_{s}+u_{w}-2u_{h}-2\beta \mathrm{\backslash rightleft}(u_{h}-u_{s}-\gamma \right)}P$ $\Delta t<\frac{\left(u_{w}-u_{s}-2\beta -2\gamma \right)}{(\beta +\gamma )\left(u_{h}-u_{w}+\beta \right)}P-\frac{2u_{h}-u_{s}-u_{w}-2\gamma }{\beta +\gamma }\frac{N}{s}$ $\Delta t\geq \frac{1}{\left(u_{s}+\gamma {-u}_{h}\right)}P+\frac{\left(-u_{h}+u_{w}-\beta \mathrm{\backslash rightleft}(2u_{h}-u_{s}-u_{w}-2\gamma \right)}{\left(u_{w}-u_{s}-2\beta -2\gamma \right)\left(u_{s}+\gamma {-u}_{h}\right)}\frac{N}{s}$ $\Delta t<\frac{1}{u_{s}+\gamma {-u}_{h}}P+\frac{u_{w}-u_{h}-\beta }{\beta +\gamma }\frac{N}{s}$	(g)

**Table 2:** Critical time points in each equilibrium travel pattern.

Patterns	Time points	Expressions
1	$t_{q}$	${t_{s}}^{*}-\frac{u_{s}+u_{w}+\gamma -2u_{h}-\beta }{A}\left(\frac{\left(u_{h}-u_{w}-\gamma \mathrm{\backslash rightleft}(u_{w}-\beta -u_{h}\right)}{\beta +\gamma }\frac{N}{s}-\left(u_{h}-u_{w}+\beta \right)\Delta t+P\right)$
	$t_{1}$	${t_{s}}^{*}-\frac{u_{s}+u_{w}-2u_{h}-2\beta }{A}\left(\frac{\left(u_{h}-u_{w}-\gamma \mathrm{\backslash rightleft}(u_{w}-\beta -u_{h}\right)}{\beta +\gamma }\frac{N}{s}-\left(u_{h}-u_{w}+\beta \right)\Delta t+P\right)$
	$t_{2}$	${t_{w}}^{*}-\frac{u_{w}+\gamma -u_{h}}{A}\left(\frac{\left({2u}_{h}-u_{s}-u_{w}+2\beta \mathrm{\backslash rightleft}(u_{s}+u_{w}+\gamma -2u_{h}-\beta \right)}{\beta +\gamma }\frac{N}{s}+\left(u_{h}-u_{w}+\beta \right)\Delta t-P\right)$
	$t_{q^{\prime }}$	${t_{w}}^{*}-\frac{u_{w}-\beta -u_{h}}{A}\left(\frac{\left({2u}_{h}-u_{s}-u_{w}+2\beta \mathrm{\backslash rightleft}(u_{s}+u_{w}+\gamma -2u_{h}-\beta \right)}{\beta +\gamma }\frac{N}{s}+\left(u_{h}-u_{w}+\beta \right)\Delta t-P\right)$
2	$t_{q}$	$\frac{u_{h}-u_{w}-\gamma }{u_{h}-u_{s}+2\beta +\gamma }\frac{N}{s}+\frac{\beta +\gamma }{u_{h}-u_{s}+2\beta +\gamma }{t_{w}}^{*}+\frac{u_{h}-u_{s}+\beta }{u_{h}-u_{s}+2\beta +\gamma }{t_{s}}^{*}-\frac{1}{u_{h}-u_{s}+2\beta +\gamma }P$
	$t_{1}$	$\frac{\left(u_{w}-u_{h}+\gamma \mathrm{\backslash rightleft}(2u_{h}+2\beta -u_{s}-u_{w}\right)}{\left(u_{w}-u_{s}-\beta -\gamma \right)\left(u_{h}-u_{s}+2\beta +\gamma \right)}\frac{N}{s}-\frac{(\beta +\gamma )\left(2u_{h}-u_{s}-u_{w}+2\beta \right)}{\left(u_{w}-u_{s}-\beta -\gamma \right)\left(u_{h}-u_{s}+2\beta +\gamma \right)}{t_{w}}^{*}+\frac{\left(u_{w}-u_{s}\right)\left(u_{h}-u_{s}+\beta \right)+(\beta +\gamma )\left(u_{h}-u_{s}-\gamma \right)}{\left(u_{w}-u_{s}-\beta -\gamma \right)\left(u_{h}-u_{s}+2\beta +\gamma \right)}{t_{s}}^{*}-\frac{\left(u_{w}-u_{s}+2\beta +2\gamma \right)}{\left(u_{w}-u_{s}-\beta -\gamma \right)\left(u_{h}-u_{s}+2\beta +\gamma \right)}P$
	$t_{q^{\prime }}$	$\frac{2u_{h}-u_{s}-u_{w}+2\beta }{u_{h}-u_{s}+2\beta +\gamma }\frac{N}{s}+\frac{\beta +\gamma }{u_{h}-u_{s}+2\beta +\gamma }{t_{w}}^{*}+\frac{u_{h}-u_{s}+\beta }{u_{h}-u_{s}+2\beta +\gamma }{t_{s}}^{*}-\frac{1}{u_{h}-u_{s}+2\beta +\gamma }P$
3	$t_{q}$	$\frac{2u_{h}-u_{s}-u_{w}-2\gamma }{2(\beta +\gamma )}\frac{N}{s}+\frac{1}{2}\left({t_{w}}^{*}+{t_{s}}^{*}\right)$
	$t_{1}$	$\frac{\left({2u}_{h}-u_{s}-u_{w}+2\beta \mathrm{\backslash rightleft}(2u_{h}-u_{s}-u_{w}-2\gamma \right)}{2(\beta +\gamma )\left(u_{s}-u_{w}+\beta +\gamma \right)}\frac{N}{s}+\frac{{-2u}_{h}+3u_{s}-u_{w}+2\gamma }{2\left(u_{s}-u_{w}+\beta +\gamma \right)}{t_{s}}^{*}+\frac{{2u}_{h}-u_{s}-u_{w}+2\beta }{2\left(u_{s}-u_{w}+\beta +\gamma \right)}{t_{w}}^{*}+\frac{2}{u_{s}-u_{w}+\beta +\gamma }P$
	$t_{2}$	$\frac{\left({2u}_{h}-u_{s}-u_{w}+2\beta \mathrm{\backslash rightleft}(2u_{h}-u_{s}-u_{w}-2\gamma \right)}{2(\beta +\gamma )\left(u_{s}-u_{w}\right)}\frac{N}{s}+\frac{\left({-2u}_{h}+3u_{s}-u_{w}+2\gamma \right)}{2\left(u_{s}-u_{w}\right)}{t_{s}}^{*}+\frac{{2u}_{h}-u_{s}-u_{w}-2\gamma }{2\left(u_{s}-u_{w}\right)}{t_{w}}^{*}+\frac{2}{u_{s}-u_{w}}P$
	$t_{q^{\prime }}$	$\frac{2u_{h}-u_{s}-u_{w}+2\beta }{2(\beta +\gamma )}\frac{N}{s}+\frac{1}{2}\left({t_{w}}^{*}+{t_{s}}^{*}\right)$
4	$t_{q}$	$-\frac{\beta +\gamma }{u_{h}-u_{s}-\beta -2\gamma }{t_{w}}^{*}+\frac{u_{h}-u_{s}-\gamma }{u_{h}-u_{s}-\beta -2\gamma }{t_{s}}^{*}-\frac{2u_{h}-u_{s}-u_{w}-2\gamma }{u_{h}-u_{s}-\beta -2\gamma }\frac{N}{s}-\frac{1}{u_{h}-u_{s}-\beta -2\gamma }P$
	$t_{1}$	$-\frac{\left({-u_{h}+u}_{w}-\beta \right)(\beta +\gamma )}{\left(u_{w}-u_{s}\mathrm{\backslash rightleft}(u_{h}-u_{s}-\beta -2\gamma \right)}{t_{w}}^{*}+\frac{\left(u_{w}-u_{s}\right)\left(u_{h}-u_{s}-\gamma \right)+(\beta +\gamma )\left(-u_{h}+u_{s}-\beta \right)}{\left(u_{w}-u_{s}\right)\left(u_{h}-u_{s}-\beta -2\gamma \right)}{t_{s}}^{*}-\frac{\left({-u_{h}+u}_{w}-\beta \right)\left(2u_{h}-u_{s}-u_{w}-2\gamma \right)}{\left(u_{w}-u_{s}\right)\left(u_{h}-u_{s}-\beta -2\gamma \right)}\frac{N}{s}-\frac{u_{w}-u_{s}-2\beta -2\gamma }{\left(u_{w}-u_{s}\right)\left(u_{h}-u_{s}-\beta -2\gamma \right)}P$
	$t_{2}$	$-\frac{\left(-u_{h}+u_{w}-\beta \right)(\beta +\gamma )}{\left(u_{w}-u_{s}-\beta -\gamma \mathrm{\backslash rightleft}(u_{h}-u_{s}-\beta -2\gamma \right)}{t_{w}}^{*}+\frac{\left(u_{w}-u_{s}-2\beta -2\gamma \right)\left(u_{h}-u_{s}-\gamma \right)}{\left(u_{w}-u_{s}-\beta -\gamma \right)\left(u_{h}-u_{s}-\beta -2\gamma \right)}{t_{s}}^{*}-\frac{\left({-u_{h}+u}_{w}-\beta \right)\left(2u_{h}-u_{s}-u_{w}-2\gamma \right)}{\left(u_{w}-u_{s}-\beta -\gamma \right)\left(u_{h}-u_{s}-\beta -2\gamma \right)}\frac{N}{s}-\frac{u_{w}-u_{s}-2\beta -2\gamma }{\left(u_{w}-u_{s}-\beta -\gamma \right)\left(u_{h}-u_{s}-\beta -2\gamma \right)}P$
	$t_{3}$	$-\frac{\left(-2u_{h}+u_{w}+u_{s}+2\gamma \right)(\beta +\gamma )}{\left(u_{w}-u_{s}\mathrm{\backslash rightleft}(u_{h}-u_{s}-\beta -2\gamma \right)}{t_{w}}^{*}+\frac{\left(u_{w}-u_{s}-2\beta -2\gamma \right)\left(u_{h}-u_{s}-\gamma \right)}{\left(u_{w}-u_{s}\right)\left(u_{h}-u_{s}-\beta -2\gamma \right)}{t_{s}}^{*}-\frac{\left(-u_{h}+u_{w}-\beta \right)\left(2u_{h}-u_{s}-u_{w}-2\gamma \right)}{\left(u_{w}-u_{s}\right)\left(u_{h}-u_{s}-\beta -2\gamma \right)}\frac{N}{s}-\frac{u_{w}-u_{s}-2\beta -2\gamma }{\left(u_{w}-u_{s}\right)\left(u_{h}-u_{s}-\beta -2\gamma \right)}P$
	$t_{q^{\prime }}$	$-\frac{\beta +\gamma }{u_{h}-u_{s}-\beta -2\gamma }{t_{w}}^{*}+\frac{u_{h}-u_{s}-\gamma }{u_{h}-u_{s}-\beta -2\gamma }{t_{s}}^{*}-\frac{u_{h}-u_{w}+\beta }{u_{h}-u_{s}-\beta -2\gamma }\frac{N}{s}-\frac{1}{u_{h}-u_{s}-\beta -2\gamma }P$
5	$t_{q}$	$\frac{u_{h}-u_{w}-\gamma }{\beta +\gamma }\frac{N}{s}+{t_{w}}^{*}$
	$t_{1}$	$-\frac{\left(u_{h}-u_{w}+\beta \mathrm{\backslash rightleft}(u_{h}-u_{w}-\gamma \right)}{(\beta +\gamma )\left(u_{w}-u_{s}\right)}\frac{N}{s}-\frac{u_{h}-u_{w}+\beta }{u_{w}-u_{s}}{t_{w}}^{*}+\frac{u_{h}-u_{s}+\beta }{u_{w}-u_{s}}{t_{s}}^{*}-\frac{1}{u_{w}-u_{s}}P$
	$t_{2}$	$-\frac{\left(u_{h}-u_{w}+\beta \mathrm{\backslash rightleft}(u_{h}-u_{w}-\gamma \right)}{(\beta +\gamma )\left(u_{w}-u_{s}-\beta -\gamma \right)}\frac{N}{s}-\frac{u_{h}-u_{w}+\beta }{u_{w}-u_{s}-\beta -\gamma }{t_{w}}^{*}+\frac{u_{h}-u_{s}-\gamma }{u_{w}-u_{s}-\beta -\gamma }{t_{s}}^{*}-\frac{1}{u_{w}-u_{s}-\beta -\gamma }P$
	$t_{q^{\prime }}$	$\frac{u_{h}-u_{w}+\beta }{\beta +\gamma }\frac{N}{s}+{t_{w}}^{*}$
6	$t_{q}$	$-\frac{2u_{h}-u_{s}-u_{w}-2\gamma }{u_{h}-u_{s}-\beta -2\gamma }\frac{N}{s}-\frac{\beta +\gamma }{u_{h}-u_{s}-\beta -2\gamma }{t_{w}}^{*}+\frac{u_{h}-u_{s}-\gamma }{u_{h}-u_{s}-\beta -2\gamma }{t_{s}}^{*}-\frac{1}{u_{h}-u_{s}-\beta -2\gamma }P$
	$t_{1}$	$\frac{\left(2u_{h}-u_{s}-u_{w}-2\gamma \mathrm{\backslash rightleft}(u_{h}-u_{w}+\beta \right)}{\left(u_{w}-u_{s}\right)\left(u_{h}-u_{s}-\beta -2\gamma \right)}\frac{N}{s}+\frac{(\beta +\gamma )\left(u_{h}-u_{w}+\beta \right)}{\left(u_{w}-u_{s}\right)\left(u_{h}-u_{s}-\beta -2\gamma \right)}{t_{w}}^{*}+\frac{\left(u_{w}-u_{s}\right)\left(u_{h}-u_{s}-\gamma \right)-(\beta +\gamma )\left(u_{h}-u_{s}+\beta \right)}{\left(u_{w}-u_{s}\right)\left(u_{h}-u_{s}-\beta -2\gamma \right)}{t_{s}}^{*}+\frac{\left(2\beta +2\gamma -u_{w}+u_{s}\right)}{\left(u_{w}-u_{s}\right)\left(u_{h}-u_{s}-\beta -2\gamma \right)}P$
	$t_{q^{\prime }}$	$\frac{{u_{w}-u}_{h}-\beta }{u_{h}-u_{s}-\beta -2\gamma }\frac{N}{s}-\frac{\beta +\gamma }{u_{h}-u_{s}-\beta -2\gamma }{t_{w}}^{*}+\frac{u_{h}-u_{s}-\gamma }{u_{h}-u_{s}-\beta -2\gamma }{t_{s}}^{*}-\frac{1}{u_{h}-u_{s}-\beta -2\gamma }P$
7	$t_{q}$	$-\frac{2u_{h}-u_{s}-u_{w}-2\gamma }{u_{h}-u_{s}-\beta -2\gamma }\frac{N}{s}-\frac{\beta +\gamma }{u_{h}-u_{s}-\beta -2\gamma }{t_{w}}^{*}+\frac{u_{h}-u_{s}-\gamma }{u_{h}-u_{s}-\beta -2\gamma }{t_{s}}^{*}-\frac{1}{u_{h}-u_{s}-\beta -2\gamma }P$
	$t_{1}$	$-\frac{\left(2u_{h}-u_{s}-u_{w}-2\gamma \mathrm{\backslash rightleft}(u_{h}-u_{w}+\beta \right)}{\left(u_{h}-u_{s}-\beta -2\gamma \right)\left(u_{s}-u_{w}\right)}\frac{N}{s}-\frac{(\beta +\gamma )\left(2u_{h}-u_{s}-u_{w}-2\gamma \right)}{\left(u_{h}-u_{s}-\beta -2\gamma \right)\left(u_{s}-u_{w}\right)}{t_{w}}^{*}+\frac{\left(u_{h}-u_{s}-\gamma \right)\left(u_{s}-u_{w}+2\beta +2\gamma \right)}{\left(u_{h}-u_{s}-\beta -2\gamma \right)\left(u_{s}-u_{w}\right)}{t_{s}}^{*}-\frac{\left(u_{s}-u_{w}+2\beta +2\gamma \right)}{\left(u_{s}-u_{w}\right)\left(u_{h}-u_{s}-\beta -2\gamma \right)}P$
	$t_{q^{\prime }}$	$\frac{{u_{w}-u}_{h}-\beta }{u_{h}-u_{s}-\beta -2\gamma }\frac{N}{s}-\frac{\beta +\gamma }{u_{h}-u_{s}-\beta -2\gamma }{t_{w}}^{*}+\frac{u_{h}-u_{s}-\gamma }{u_{h}-u_{s}-\beta -2\gamma }{t_{s}}^{*}-\frac{1}{u_{h}-u_{s}-\beta -2\gamma }P$

Notes: $A=\left(u_{h}-u_{w}-\gamma \mathrm{\backslash rightleft}(u_{w}-\beta -u_{h}\right)+\left({2u}_{h}-u_{s}-u_{w}+2\beta \right)\left(u_{s}+u_{w}+\gamma -2u_{h}-\beta \right)$*.*

Pattern 1: In this case, Δt is relatively large. Household commuters in Group 1 depart near school start time ${t_{s}}^{*}$, while those in Group 2 depart near work start time ${t_{w}}^{*}$. As a result, the travels of the two groups are completely separated.

Pattern 2: In this case, Δt is smaller than that in Pattern 1. For two groups of household commuters, the travels are connected, which means that the first household commuter of Group 2 will join the queue behind the last household commuter of Group 1.

Pattern 3: In this case, Δt is relatively small compared to that in Pattern 2, and school bus fare P is high. Due to the high cost, most household commuters choose to drive their children to school by themselves. As shown in Fig 2(c), the household commuters of Group 2 depart near ${\tilde{t}}_{w}$ to maximize their net utilities. Since the number of commuters in Group 1 is relatively large, some commuters of Group 1 are forced to depart after the commuters of Group 2.

Pattern 4: In this case, school bus fare P is moderate, which means that a similar number of household commuters are in two groups. As shown in Fig 2, the departure times of the two groups of household commuters are interleaved with each other. Due to the impact of the school utility, in Group 1, most of the household commuters depart near time ${\tilde{t}}_{s}$. Due to the impact of the work trip utility, in Group 2, most of the household commuters depart near time ${\tilde{t}}_{w}$. In Group 1, the small number of household commuters are forced to leave at the end of morning peak hours, while, in group 2, the small number of household commuters are forced to leave at the beginning of morning peak hours.

Pattern 5: Similar to Pattern 3 and Pattern 4, Δt is small. However, P is very low in this case, which means that most children go to school by school bus, resulting in a relatively large number of household commuters in Group 2. Therefore, some commuters of Group 2 are forced to leave before all household commuters of Group 1, as shown in Fig 2(e).

Pattern 6: In this case, Δt is the smallest compared to Pattern 3–5, and P is high. This results in the relatively large number of household commuters in Group 1. As shown in Fig 2(f), all household commuters of Group 2 depart before those in Group 1 to arrive at workplace early.

Pattern 7: In contrast to Pattern 6, school bus fare P is low, which leads to the relatively large number of household commuters in Group 2. As shown in Fig 2(g), all household commuters in Group 1 depart after those in Group 2 and arrive at workplace later than those in Group 2.

**Fig 3:**
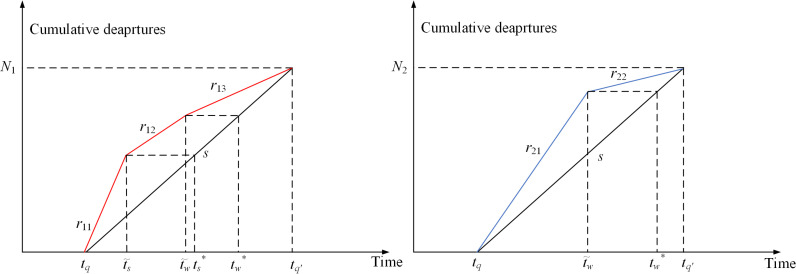
Extreme cases. (a) Extreme case i. (b) Extreme case ii.

Based on an assumption that the number of household commuters in both groups is positive, all possible equilibrium travel patterns have been analyzed. However, due to the impact of school bus fare, two extreme cases exist, i.e., one of the two groups of commuters disappeared, as depicted in Fig 3. Fig 3(a) shows an extreme scenario (i) in which all household commuters drop off their children at school before heading to work. In contrast, Fig 3(b) shows another extreme scenario (ii) in which all children go to school by school bus, while all adults drive to work. Table 3 summarizes the occurrence of these two extreme cases in all possible equilibrium travel patterns. Both two extreme cases are in Pattern 1 and Pattern 2, only one extreme case is in Pattern 3, Pattern 5, Pattern 6 and Pattern 7, and no extreme case is in Pattern 4. This is because, as the school bus fare increases or decreases, the equilibrium travel pattern will remain unchanged in Pattern 1 and Pattern 2, while other patterns will change to another pattern. For example, in Pattern 3, when P increases to $P=\frac{\left({2u}_{h}-u_{s}-u_{w}+2\beta \mathrm{\backslash rightleft}(u_{s}+u_{w}+2\gamma -2u_{h}\right)}{4(\beta +\gamma )}\frac{N}{s}+\frac{{-2u}_{h}+3u_{s}-u_{w}+2\gamma }{4}\Delta t$, the household commuters of Group 2 will vanish. This means that all household commuters will drop off their children at school before heading to work. However, when P decreases to $P=\frac{\left(u_{s}+u_{w}+2\gamma -2u_{h}\mathrm{\backslash rightleft}(u_{h}-u_{s}+\beta \right)}{2(\beta +\gamma )}\frac{N}{s}-\frac{\left(u_{h}-u_{s}+\beta \right)}{2}\Delta t$, the equilibrium travel pattern will change from Pattern 3 to Pattern 4. In Pattern 4, as P continues to decrease to $P=\left(u_{s}+\gamma -u_{h}\right)\Delta t-\frac{\left(u_{w}-u_{h}-\beta \mathrm{\backslash rightleft}(u_{s}+\gamma -u_{h}\right)}{\beta +\gamma }\frac{N}{s}$ (or $P=\left(u_{s}+\gamma -u_{h}\right)\Delta t-\frac{\left(u_{w}-u_{h}-\beta \mathrm{\backslash rightleft}(u_{s}+\gamma -u_{h}\right)}{\beta +\gamma }\frac{N}{s}$), the equilibrium travel pattern will change from Pattern 4 to Pattern 5 (or Pattern 7). From the above analysis, it can infer that Pattern 3 has only one extreme case, while Pattern 4 has no extreme case.

**Table 3:** The occurrence conditions for extreme cases.

Patterns	Conditions	Extreme cases
1	$P\geq \frac{\left({2u}_{h}-u_{s}-u_{w}+2\beta \mathrm{\backslash rightleft}(u_{s}+u_{w}+\gamma -2u_{h}-\beta \right)}{\beta +\gamma }\frac{N}{s}+\left(u_{h}-u_{w}+\beta \right)\Delta t$	(i)
	$P\leq \frac{-\left(u_{h}-u_{w}-\gamma \mathrm{\backslash rightleft}(u_{w}-\beta -u_{h}\right)}{\beta +\gamma }\frac{N}{s}+\left(u_{h}-u_{w}+\beta \right)\Delta t$	(ii)
2	$P\geq \frac{\left(u_{s}+\beta +2\gamma -u_{h}\mathrm{\backslash rightleft}(2u_{h}+2\beta -u_{s}-u_{w}\right)}{3(\beta +\gamma )}\frac{N}{s}-\frac{\left(2u_{h}-2u_{s}+\beta -\gamma \right)}{3}\Delta t$	(i)
	$P\leq \frac{\left({u_{w}+\gamma -u}_{h}\mathrm{\backslash rightleft}(2u_{h}+\beta -{2u}_{s}-\gamma \right)}{3(\beta +\gamma )}\frac{N}{s}-\frac{\left(2u_{h}-2u_{s}+\beta -\gamma \right)}{3}\Delta t$	(ii)
3	$P\geq \frac{\left({2u}_{h}-u_{s}-u_{w}+2\beta \mathrm{\backslash rightleft}(u_{s}+u_{w}+2\gamma -2u_{h}\right)}{4(\beta +\gamma )}\frac{N}{s}+\frac{{-2u}_{h}+3u_{s}-u_{w}+2\gamma }{4}\Delta t$	(i)
4	−	−
5	$P\leq \frac{\left(u_{h}-u_{w}+\beta \mathrm{\backslash rightleft}({u_{w}+\gamma -u}_{h}\right)}{\beta +\gamma }\frac{N}{s}-\left(u_{h}-u_{w}+\beta \right)\Delta t$	(ii)
6	$P\geq \frac{\left(u_{s}+u_{w}+2\gamma -2u_{h}\mathrm{\backslash rightleft}(u_{h}-u_{s}+\beta \right)}{2(\beta +\gamma )}\frac{N}{s}-\frac{u_{h}-u_{s}+\beta }{2}\Delta t$	(i)
7	$P\leq \frac{\left(\gamma +u_{s}-u_{h}\mathrm{\backslash rightleft}(u_{h}-u_{w}+\beta \right)}{\left(\beta +\gamma \right)}\frac{N}{s}+\left(\gamma +u_{s}-u_{h}\right)\Delta t$	(ii)s

### User net utility

This section examines the influence of school bus fare P, school start time $t_{s}^{*}$ and work start time $t_{w}^{*}$ on user net utility ψ. At the equilibrium, all household commuters have the same net utility. According to the analysis in Section 2: Problem description and model formulation, net utility of household commuters for each equilibrium travel pattern is calculated, and more details are listed in Table 4.

**Table 4:** User net utility.

Patterns	User net utility
1	$-\frac{\left(u_{h}-u_{w}-\gamma \mathrm{\backslash rightleft}(u_{w}-\beta -u_{h}\right)\left({2u}_{h}-u_{s}-u_{w}+2\beta \right)\left(u_{s}+u_{w}+\gamma -2u_{h}-\beta \right)}{(\beta +\gamma )A}\frac{N}{s}-\frac{\beta \left(u_{h}-u_{w}-\gamma \right)\left(u_{w}-\beta -u_{h}\right)-\left(u_{h}-u_{w}\right)\left({2u}_{h}-u_{s}-u_{w}+2\beta \right)\left(u_{s}+u_{w}+\gamma -2u_{h}-\beta \right)}{A}{t_{w}}^{*}+\frac{\left({2u}_{h}-u_{w}-u_{s}+\beta \right)\left(u_{h}-u_{w}-\gamma \right)\left(u_{w}-\beta -u_{h}\right)+\left(u_{h}-u_{s}\right)\left({2u}_{h}-u_{s}-u_{w}+2\beta \right)\left(u_{s}+u_{w}+\gamma -2u_{h}-\beta \right)}{A}{t_{s}}^{*}-\frac{\left({2u}_{h}-u_{s}-u_{w}+2\beta \right)\left(u_{s}+u_{w}+\gamma -2u_{h}-\beta \right)}{A}P-2u_{h}t_{-}+\left(u_{s}+u_{w}\right)t^{-}$
2	$\frac{\left(u_{h}-u_{w}-\gamma \mathrm{\backslash rightleft}({2u}_{h}-u_{s}-u_{w}+2\beta \right)}{u_{h}-u_{s}+2\beta +\gamma }\frac{N}{s}+\frac{\beta \left(u_{h}-u_{w}\right)+\gamma \left({2u}_{h}-u_{s}-u_{w}+\beta \right)}{u_{h}-u_{s}+2\beta +\gamma }{t_{w}}^{*}+\frac{\left(u_{h}-u_{s}\right)\left({2u}_{h}-u_{s}-u_{w}+2\beta \right)+\beta \left(u_{h}-u_{w}-\gamma \right)}{u_{h}-u_{s}+2\beta +\gamma }{t_{s}}^{*}-\frac{\left({2u}_{h}-u_{s}-u_{w}+2\beta \right)}{u_{h}-u_{s}+2\beta +\gamma }P-2u_{h}t_{-}+\left(u_{s}+u_{w}\right)t^{-}$
3	$\frac{\left({2u}_{h}-u_{s}-u_{w}+2\beta \mathrm{\backslash rightleft}(2u_{h}-u_{s}-u_{w}-2\gamma \right)}{2(\beta +\gamma )}\frac{N}{s}+\frac{\left({2u}_{h}-u_{s}-u_{w}\right)}{2}\left({t_{w}}^{*}+{t_{s}}^{*}\right)-2u_{h}t_{-}+\left(u_{s}+u_{w}\right)t^{-}$
4	$-\frac{\left(u_{h}-u_{w}+\beta \mathrm{\backslash rightleft}(2u_{h}-u_{s}-u_{w}-2\gamma \right)}{\left(u_{h}-u_{s}-\beta -2\gamma \right)}\frac{N}{s}-\frac{\gamma \left(u_{h}-u_{w}-\beta \right)+\beta \left(2u_{h}-u_{w}-u_{s}\right)}{\left(u_{h}-u_{s}-\beta -2\gamma \right)}{t_{w}}^{*}+\frac{\left(u_{h}-u_{s}\right)\left(2u_{h}-u_{w}-u_{s}-2\gamma \right)-\left(u_{h}-u_{w}+\beta \right)\gamma }{\left(u_{h}-u_{s}-\beta -2\gamma \right)}{t_{s}}^{*}-\frac{\left(2u_{h}-u_{s}-u_{w}-2\gamma \right)}{\left(u_{h}-u_{s}-\beta -2\gamma \right)}P-{2u}_{h}t_{-}+\left(u_{s}+u_{w}\right)t^{-}$
5	$\frac{\left(u_{h}-u_{w}+\beta \mathrm{\backslash rightleft}(u_{h}-u_{w}-\gamma \right)}{\beta +\gamma }\frac{N}{s}+\left(u_{h}-u_{w}\right){t_{w}}^{*}+\left(u_{h}-u_{s}\right){t_{s}}^{*}-P-2u_{h}t_{-}+\left(u_{s}+u_{w}\right)t^{-}$
6	$-\frac{\left(u_{h}-u_{w}+\beta \mathrm{\backslash rightleft}(2u_{h}-u_{s}-u_{w}-2\gamma \right)}{\left(u_{h}-u_{s}-\beta -2\gamma \right)}\frac{N}{s}-\frac{\gamma \left(u_{h}-u_{w}-\beta \right)+\beta \left(2u_{h}-u_{w}-u_{s}\right)}{\left(u_{h}-u_{s}-\beta -2\gamma \right)}{t_{w}}^{*}+\frac{\left(u_{h}-u_{s}\right)\left(2u_{h}-u_{w}-u_{s}-2\gamma \right)-\left(u_{h}-u_{w}+\beta \right)\gamma }{\left(u_{h}-u_{s}-\beta -2\gamma \right)}{t_{s}}^{*}-\frac{\left(2u_{h}-u_{s}-u_{w}-2\gamma \right)}{\left(u_{h}-u_{s}-\beta -2\gamma \right)}P-2u_{h}t_{-}+\left(u_{s}+u_{w}\right)t^{-}$
7	$-\frac{\left(u_{h}-u_{w}+\beta \mathrm{\backslash rightleft}(2u_{h}-u_{s}-u_{w}-2\gamma \right)}{\left(u_{h}-u_{s}-\beta -2\gamma \right)}\frac{N}{s}-\frac{\gamma \left(u_{h}-u_{w}-\beta \right)+\beta \left(2u_{h}-u_{w}-u_{s}\right)}{\left(u_{h}-u_{s}-\beta -2\gamma \right)}{t_{w}}^{*}+\frac{\left(u_{h}-u_{s}\right)\left(2u_{h}-u_{w}-u_{s}-2\gamma \right)-\left(u_{h}-u_{w}+\beta \right)\gamma }{\left(u_{h}-u_{s}-\beta -2\gamma \right)}{t_{s}}^{*}-\frac{\left(2u_{h}-u_{s}-u_{w}-2\gamma \right)}{\left(u_{h}-u_{s}-\beta -2\gamma \right)}P-2u_{h}t_{-}+\left(u_{s}+u_{w}\right)t^{-}$

From Table 4, the following propositions are concluded.

**Proposition 3**. For given school start time $t_{s}^{*}$ and work start time $t_{w}^{*}$, it can be obtained as follows:


$$\frac{d\psi }{dP}\leq 0.$$ (28)

**Proof**: The validity of Proposition 3 can be readily confirmed by referencing Table 4.

As Proposition 3 highlights, for given school start time $t_{s}^{*}$ and work start time $t_{w}^{*}$, a reduction in school bus fare proves advantageous to all household commuters.

**Proposition 4**. For given school bus fare P and work start time $t_{w}^{*}$, we have:

(1) The following inequality is established in Pattern 2, Pattern 3 and Pattern 5:


$$\frac{d\psi }{dt_{s}^{*}}<0.$$ (29)

(2) In Pattern 1, two situations exist:

①When$\left({2u}_{h}-u_{w}-u_{s}+\beta \mathrm{\backslash rightleft}(u_{h}-u_{w}-\gamma \right)\left(u_{w}-\beta -u_{h}\right)<\left(u_{s}-u_{h}\right)\left({2u}_{h}-u_{s}-u_{w}+2\beta \right)\left(u_{s}+u_{w}+\gamma -2u_{h}-\beta \right)$, we have:


$$\frac{d\psi }{dt_{s}^{*}}<0;$$ (30)

②When $\left({2u}_{h}-u_{w}-u_{s}+\beta \mathrm{\backslash rightleft}(u_{h}-u_{w}-\gamma \right)\left(u_{w}-\beta -u_{h}\right)\geq \left(u_{s}-u_{h}\right)\left({2u}_{h}-u_{s}-u_{w}+2\beta \right)\left(u_{s}+u_{w}+\gamma -2u_{h}-\beta \right)$, we have:


$$\frac{d\psi }{dt_{s}^{*}}\geq 0.$$ (31)

(3) In pattern 4, pattern 6 and pattern 7, two situations exist:

① When $\left(u_{h}-u_{s}\mathrm{\backslash rightleft}(2u_{h}-u_{w}-u_{s}-2\gamma \right)>\left(u_{h}-u_{w}+\beta \right)\gamma$, we have:


$$\frac{d\psi }{dt_{s}^{*}}<0;$$ (32)

② When $\left(u_{h}-u_{s}\mathrm{\backslash rightleft}(2u_{h}-u_{w}-u_{s}-2\gamma \right)\leq \left(u_{h}-u_{w}+\beta \right)\gamma$, we have:


$$\frac{d\psi }{dt_{s}^{*}}\geq 0.$$ (33)

**Proof**: This proposition can be easily proved by referring to Table 4.

**Proposition 5**. For given school bus fare P and school start time $t_{s}^{*}$, we have:

(1) In Pattern 1, 3, 4, 5, 6 and 7, the following inequality is established:


$$\frac{d\psi }{d{t_{w}}^{*}}<0.$$ (34)

(2) In Pattern 2, two situations exist:

① When $\beta \left(u_{w}-u_{h}\right)>\gamma \left({2u}_{h}-u_{s}-u_{w}+\beta \right)$, we have:


$$\frac{d\psi }{d{t_{w}}^{*}}<0;$$ (35)

② When $\beta \left(u_{w}-u_{h}\right)\leq \gamma \left({2u}_{h}-u_{s}-u_{w}+\beta \right)$, we have


$$\frac{d\psi }{d{t_{w}}^{*}}\geq 0.$$ (36)

**Proof**: This proposition can be easily proved by referring to Table 4.

Proposition 4 and 5 indicate that, in different patterns, due to the impact of activity utility and schedule delay, the user net utility varies differently with changes in $t_{w}^{*}$ or $t_{s}^{*}$. Therefore, to increase the net utility of household commuters by adjusting school or work start time, the impact of activity utility and schedule delay must both be considered. For example, when $\beta \left(u_{w}-u_{h}\right)>\gamma \left({2u}_{h}-u_{s}-u_{w}+\beta \right)$, advancing work start time will increase the net utility of users in Pattern 2. However, when $\beta \left(u_{w}-u_{h}\right)<\gamma \left({2u}_{h}-u_{s}-u_{w}+\beta \right)$, advancing work start time will decrease the net utility of users in Pattern 2.

### System optimum

**Fig 4:**
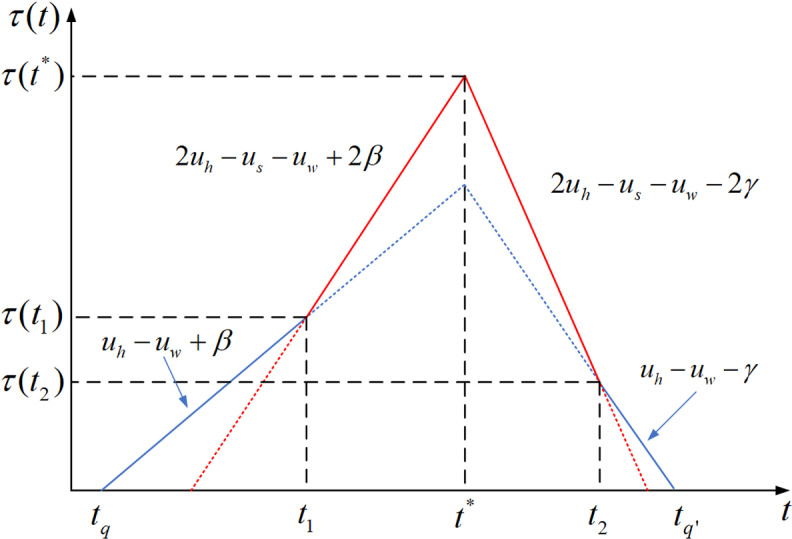
First-best time-varying toll.


This section proposes a first-best time-varying toll to manage morning household commuting problems. It is assumed that school bus fare is within a reasonable range, which means that there are two groups of household commuters in the network. In the system optimum, there should be no queuing at the bottleneck (D(t)=0), and the negative utility of household commuters should be the smallest. This can be achieved by setting Δt=0 (${{t_{s}^{*}=t}_{w}}^{*}$) and implementing a first-best time-varying toll (τ(t)) [27]. In this situation, household commuters of Group 1 need to depart near the school start time (also the work start time) because they need to consider the net utility of both adults and children. According to Eq. (9) and Eq. (14), under the first-best time-varying toll, the net utilities of both groups of household commuters can be expressed as


$$\psi_{1}(t)=\left\{ \begin{array}{c} \left({2u}_{h}-u_{s}-u_{w}+2\beta \right)t-2\beta t^{*}-{2u}_{h}t_{-}+\left(u_{s}+u_{w}\right)t^{-}-\tau (t),t<t^{*}\\ \left(2u_{h}-u_{s}-u_{w}-2\gamma \right)t+2\gamma t^{*}-{2u}_{h}t_{-}+\left(u_{s}+u_{w}\right)t^{-}-\tau (t),t>t^{*} \end{array}\right.,$$ (35)

$$\psi_{2}(t)=\left\{ \begin{array}{c} \left(u_{h}-u_{w}+\beta \right)t+\left(u_{h}-u_{s}-\beta \right)t^{*}-2u_{h}t_{-}+\left(u_{s}+u_{w}\right)t^{-}-P-\tau (t),t<t^{*}\\ \left(u_{h}-u_{w}-\gamma \right)t+\left(u_{h}-u_{s}+\gamma \right)t^{*}-2u_{h}t_{-}+\left(u_{s}+u_{w}\right)t^{-}-P-\tau (t),t\geq t^{*} \end{array},\right.$$ (36)

where school and work are the same start time, namely $t^{*}$. Given $t^{*}$ and P, according to Eq. (35) and Eq. (36), the first-best time-varying toll can be expressed as


$$\tau (t)=\left\{ \begin{array}{c} @l0,t<t_{q}\\ \left(u_{h}-u_{w}+\beta \right)t-\left(u_{h}-u_{w}+\beta \right)t^{*}+\frac{\left(u_{h}-u_{w}+\beta \right)\left(u_{w}+\gamma -u_{h}\right)}{\beta +\gamma }\frac{N}{s},t_{q}\leq t<t_{1}\\ \left({2u}_{h}-u_{s}-u_{w}+2\beta \right)t-\left(2u_{h}-u_{s}-u_{w}+2\beta \right)t^{*}+\frac{\left(u_{h}-u_{w}+\beta \right)\left(u_{w}+\gamma -u_{h}\right)}{\beta +\gamma }\frac{N}{s}+P,t_{1}\leq t_{q}<t^{*}\\ \left(2u_{h}-u_{s}-u_{w}-2\gamma \right)t-\left(2u_{h}-u_{s}-u_{w}-2\gamma \right)t^{*}+\frac{\left(u_{h}-u_{w}+\beta \right)\left(u_{w}+\gamma -u_{h}\right)}{\beta +\gamma }\frac{N}{s}+P,t^{*}\leq t<t_{2}\\ \left(u_{h}-u_{w}-\gamma \right)t+\left(u_{w}-u_{h}+\gamma \right)t^{*}+\frac{\left(u_{h}-u_{w}+\beta \right)\left(u_{w}+\gamma -u_{h}\right)}{\beta +\gamma }\frac{N}{s},t_{2}\leq t<t_{q^{\prime }}\\ 0,t\geq t_{q^{\prime }} \end{array},\right.$$ (37)

where $t_{q}=t^{*}-\frac{u_{w}+\gamma -u_{h}}{\beta +\gamma }\frac{N}{s}$, $t_{1}=t^{*}-\frac{1}{\left(u_{h}-u_{s}+\beta \right)}P$, $t_{2}=t^{*}+\frac{1}{u_{s}+\gamma -u_{h}}P$, $t_{q^{\prime }}=t^{*}+\frac{u_{h}-u_{w}+\beta }{\beta +\gamma }\frac{N}{s}$. Fig 4 illustrates the optimal toll value. As shown in Fig 4, the maximum toll occurs at time $t^{*}$. The specific value of maximum toll can be obtained by using Eq. (37)

$$\tau \left(t^{*}\right)=\frac{\left(u_{h}-u_{w}+\beta \mathrm{\backslash rightleft}(u_{w}+\gamma -u_{h}\right)}{\beta +\gamma }\frac{N}{s}+P.$$ (38)

The system total net utilities at system optimum (excluding toll) can be expressed as


$$TC=\left[\left(2u_{h}-u_{w}-u_{s}\right)t^{*}-2u_{h}t_{-}+\left(u_{s}+u_{w}\right)t^{-}\right]N-\frac{\left(u_{h}-u_{w}+\beta \mathrm{\backslash rightleft}(u_{w}+\gamma -u_{h}\right)}{2(\beta +\gamma )}\frac{N^{2}}{s}-PN.$$ (39)

Eq. (39) shows that the system total net utility at system optimum is inversely proportional to school bus fare P, that is, if school bus charge is higher, then the system total net utility will be smaller. This can also indicate that as the number of children taking school bus increases, the system total net utility also increases.

### Numerical experiments

**Fig 5:**
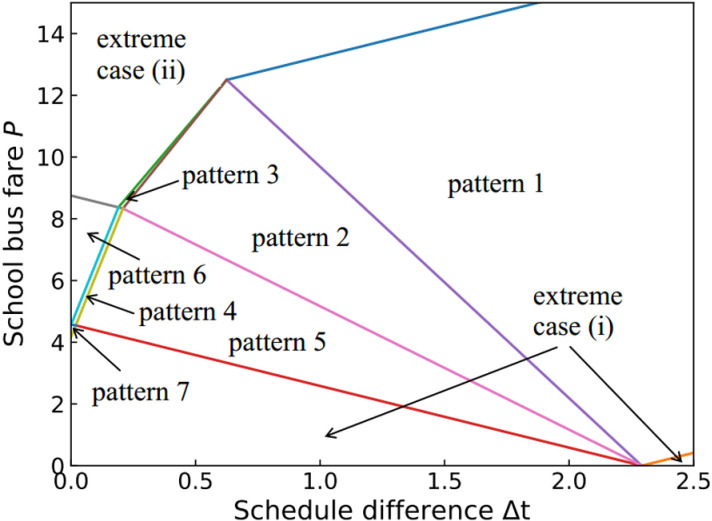
The occurrence of the different equilibrium travel patterns and the extreme cases.

This section verifies and illustrates the proposed model and the propositions by several numerical experiments. The parameters used in this experiment are listed in Table 5, based on the study by Li et al. [36], with some minor adjustments. By varying the school bus fare and the school-work start time difference, the occurrence of all equilibrium travel patterns is presented in Fig 5. The results show that seven possible equilibrium travel patterns and two extreme cases exist, which is consistent with the above analysis.

**Table 5:** Parameters in numerical experiments.

N(veh)	s(veh/h)	$t_{-}$	$t^{-}$	α (EUR$/h)	β (EUR$/h)	γ (EUR$/h)	$u_{h}$ (EUR$/h)	$u_{s}$ (EUR$/h)	$u_{w}$ (EUR$/h)
5000	2000	6:00	12:00	8	6	18	8	10	12

**Fig 6:**
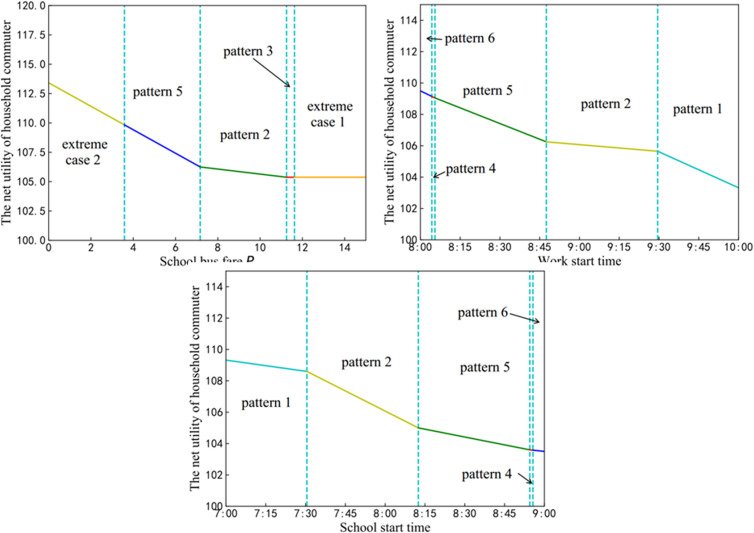
The net utility varies with school bus fare (a), work start time (b) and school start time(c).

This section studies the relationship between 3 variables (namely school bus fare, school start time and work start time) and the net utility of household commuters, respectively. Firstly, the school start time and the work start time are fixed at 8:00 and 8:30, respectively, so Δt=0.5(h). Fig 6(a) shows a trend that the net utility of household commuters varies with school bus fare. As the fare increases, the equilibrium travel pattern shifts from Pattern 5 to Pattern 2 and eventually Pattern 3. Additionally, when the fare is either very low or very high, two extreme cases exist. It is noteworthy that as the school bus fare increases, the net utility of users decreases sharply at first, then more gradually and eventually reaches a stable state. This phenomenon can be attributed to the fact that when the fare becomes sufficiently high, no children go to school by school bus, which leads to no impact on the net utility of household commuters.

Secondly, the school start time and the school bus fare are set to 8: 00 and 6 (EUR$/day), respectively. Fig 6(b) illustrates the impact of the work start time on the net utility of household commuters. The equilibrium travel pattern follows a path: Pattern 6 → Pattern 4 → Pattern 5 → Pattern 2 → Pattern 1, as the work start time is delayed. According to Proposition 5, when $\beta \left(u_{w}-u_{h}\right)=24>\gamma \left({2u}_{h}-u_{s}-u_{w}+\beta \right)=0$, the net utility of users is inversely proportional to the work start time across all possible travel patterns. Therefore, as the work start time is delayed, the net utility of users will decrease, as demonstrated in Fig 6(b).

Finally, the work start time and the school bus fare are set to 9: 00 and 6 (EUR$/day), respectively. Fig 6(c) shows the relationship between the net utility of household commuters and the school start time. Interestingly, the net utility of household commuters varies in the opposite direction to that observed in Fig 6(b), with the equilibrium travel pattern following a path: Pattern 1 → Pattern 2 → Pattern 5 → Pattern 4 → Pattern 6. As Proposition 4, when$\left({2u}_{h}-u_{w}-u_{s}+\beta \mathrm{\backslash rightleft}(u_{h}-u_{w}-\gamma \right)\left(u_{w}-\beta -u_{h}\right)=0<\left(u_{s}-u_{h}\right)\left({2u}_{h}-u_{s}-u_{w}+2\beta \right)\left(u_{s}+u_{w}+\gamma -2u_{h}-\beta \right)=216$, the school start time is delayed, which leads to a reduction in the net utility of users. This trend also evident in Fig 6(c).

To analyze the efficiency of first-best time-varying toll, both school and work start time are set to 8:00, and bus fare is set to 6 (EUR$/day). Based on Fig 5, it can be obtained that the equilibrium travel pattern without toll is in Pattern 6. Based on the user net utility of Pattern 6 in Table 4, the total net utility of system without toll as: ${TC}_{u}=N\cdot \psi_{6}=547500$(EUR$), is calculated. Using Eq. (39), the total net utility in first-best time-varying toll scenario as: ${TC}_{so}=\left[\left(2u_{h}-u_{w}-u_{s}\right)t^{*}-2u_{h}t_{-}+\left(u_{s}+u_{w}\right)t^{-}\right]N-\frac{\left(u_{h}-u_{w}+\beta \mathrm{\backslash rightleft}(u_{w}+\gamma -u_{h}\right)}{2(\beta +\gamma )}\frac{N^{2}}{s}-PN\approx 558542$ (EUR$), is also calculated. Thus, it can be concluded that the first-best time-varying toll can increase the system total net utility by 11042(EUR$).

## Conclusions

Based on activity method, this paper studies the household commuting problem in a bi-modal transportation system with autos and school buses. A bottleneck model is developed to capture the household commuting behaviors, and the user equilibrium travel patterns are analyzed. Additionally, a first-best time-varying toll is proposed to manage the household commuting. The following results are obtained: (i) the number of household commuters who use school bus is determined endogenous by school bus fare; (ii) the equilibrium travel patterns depend on the school bus fare and the school-work start time difference, and up to seven patterns may occur; (iii) a lower school bus fare results in higher net utility for household commuters; (iv) by adjusting the start time of school or work, it can increase the net utility of household commuters; (v) by implementing the first-best time-varying toll, it can eliminate traffic congestion and improve travel efficiency.

Although this study establishes an initial theoretical framework for travel mode choice, its applicability is limited by simplifications in factors such as traffic conditions, travel purposes, origin-destination variations, and route choices. Future research will focus on enhancing the precision of model and real-world applicability by incorporating more complex elements, such as traffic flow, route selection, and diverse origin-destination patterns, to better simulate and analyze travel behavior. Furthermore, we plan to gather relevant datasets, leverage field data sources, and employ more sophisticated parameter estimation techniques to improve the accuracy of model and the reliability of its conclusions. In subsequent studies, we also intend to extend the model to encompass more detailed family travel categories, including variations in school distances and the specific travel needs of family members, thereby increasing both the complexity and accuracy of the model.

## Supporting information

S1 File The data of this study. (ZIP)

S2 Appendix. (DOCX)
