# Supplementary material for: Modeling and managing household travels taking into considering of school bus
Source: PLoS One. 2025 May 29;20(5):e0324561. doi: 10.1371/journal.pone.0324561 (PMC12121786; doi:10.1371/journal.pone.0324561)
Supplement: S2 — (DOCX) [file pone.0324561.s002.docx]

# Appendix A

In Pattern 1, $\Delta t$ is relatively large. The travel of commuters from the two types of households is completely separated and does not affect each other. According to equation (9) and (14), we can know the net utility at time points $t_{q}$, $t_{1}$, $t_{2}$, and $t_{q'}$.

$\psi_{1}\left( t_{q} \right)=\left( {2u}_{h}-u_{s}-u_{w}+2\beta\right)t_{q}-\beta\left( {t_{w}}^{*}+{t_{s}}^{*} \right)-{2u}_{h}t_{-}+\left( u_{s}+u_{w} \right)t^{-}$ (A.1)

$\psi_{1}\left( t_{1} \right)=\left( {2u}_{h}-u_{s}-u_{w}+\beta-\gamma\right)t_{1}-\beta{t_{w}}^{*}+{\gamma t_{s}}^{*}-{2u}_{h}t_{-}+\left( u_{s}+u_{w} \right)t^{-}$ (A.2)

$\psi_{2}\left( t_{2} \right)=\left( u_{h}-u_{w}+\beta\right)t_{2}-\beta{t_{w}}^{*}+\left( u_{h}-u_{s} \right){t_{s}}^{*}-{2u}_{h}t_{-}+\left( u_{s}+u_{w} \right)t^{-}-P$ (A.3)

$\psi_{2}\left( t_{q'} \right)=\left( u_{h}-u_{w}-\gamma\right)t_{q'}+\gamma{t_{w}}^{*}+\left( u_{h}-u_{s} \right){t_{s}}^{*}-{2u}_{h}t_{-}+\left( u_{s}+u_{w} \right)t^{-}-P$ (A.4)

Furthermore, according to the principle of flow conservation, we have:

$N_{1}=\left( t_{1}-t_{q} \right)s$ (A.5)

$N_{2}=\left( t_{q'}-t_{2} \right)s$ (A.6)

$N=N_{1}+N_{2}$ (A.7)

Based on the equilibrium condition, which states that the net travel utility of all household commuters is equal, and the above equations, we can derive the expressions for the earliest and latest departure times for commuters from the two types of households:

$t_{q}={t_{s}}^{*}-\frac{u_{s}+u_{w}+\gamma-2u_{h}-\beta}{A}\left( \frac{\left( u_{h}-u_{w}-\gamma\right)\left( u_{w}-\beta-u_{h} \right)}{\beta+\gamma} \frac{N}{s}-\left( u_{h}-u_{w}+\beta\right)\Delta t+P \right)$ (A.8)

$t_{1}={t_{s}}^{*}- \frac{u_{s}+u_{w}-2u_{h}-2\beta}{A}\left( \frac{\left( u_{h}-u_{w}-\gamma\right)\left( u_{w}-\beta-u_{h} \right)}{\beta+\gamma} \frac{N}{s}-\left( u_{h}-u_{w}+\beta\right)\Delta t+P \right)$ (A.9)

$t_{2}={t_{w}}^{*}-\frac{u_{w}+\gamma-u_{h}}{A}\left( \frac{\left( {2u}_{h}-u_{s}-u_{w}+2\beta\right)\left( u_{s}+u_{w}+\gamma-2u_{h}-\beta\right)}{\beta+\gamma} \frac{N}{s}+\left( u_{h}-u_{w}+\beta\right)\Delta t-P \right)$ (A.10)

$t_{q'}={t_{w}}^{*}-\frac{u_{w}-\beta-u_{h}}{A}\left( \frac{\left( {2u}_{h}-u_{s}-u_{w}+2\beta\right)\left( u_{s}+u_{w}+\gamma-2u_{h}-\beta\right)}{\beta+\gamma} \frac{N}{s}+\left( u_{h}-u_{w}+\beta\right)\Delta t-P \right)$ (A.11)

Following a similar derivation process as in Pattern 1, we can derive all time points in each pattern, as show in Table 2. Following these time points, we can derive the occurrence condition for each pattern.

For example, in Pattern 1, based on the above analysis and formulas, we find that its occurrence requires satisfying condition $t_{1}\leq t_{2}$, where $t_{1}$ and $t_{2}$ belong to Pattern 1. Therefore, according to Table 2, the occurrence condition for Pattern 1 can be expressed as follows:

$t_{1}\leq t_{2}\Longrightarrow\Delta t\geq\frac{{\gamma-u}_{h}+u_{w}}{\beta+\gamma}\frac{N}{s}-\frac{u_{s}+2u_{w}-3u_{h}-2\beta+\gamma}{\left( u_{s}+u_{w}-2u_{h}-2\beta\right)\left( u_{h}-u_{s}-\gamma\right)}P$ (A.12)

Where $\Delta t={t_{w}}^{*}-{t_{s}}^{*}$.

For Pattern 2, it is critical with Patterns 1, 3, and 5. Therefore, based on the relevant time points expressions of Patterns 1, 3, and 5, the occurrence condition for Pattern 2 can be derived. Specifically, $t_{1}>t_{2}$ in Pattern 1, $t_{2}>t_{q'}$ in Pattern 3, and $t_{1}>t_{q}$ in Pattern 5. According to the expression of time points in each Pattern, the occurrence conditions for Pattern 2 can be derived as follows:

$\Delta t<\frac{{\gamma-u}_{h}+u_{w}}{\beta+\gamma}\frac{N}{s}-\frac{u_{s}+2u_{w}-3u_{h}-2\beta+\gamma}{\left( u_{s}+u_{w}-2u_{h}-2\beta\right)\left( u_{h}-u_{s}-\gamma\right)}P$ (A.13)

$\Delta t \geq-\frac{{2u}_{h}-u_{s}-u_{w}+2\beta}{\beta+\gamma}\frac{N}{s}+\frac{2}{{u_{s}+\gamma-u}_{h}}P$ (A.14)

$\Delta t\geq\frac{u_{w}+\gamma-u_{h}}{\beta+\gamma}\frac{N}{s}-\frac{1}{u_{h}-u_{s}+\beta}P$ (A.15)

The occurrence conditions of other travel patterns can be proven in a similar manner.
